# Role of RNA Interference (RNAi) in the Moss *Physcomitrella patens*

**DOI:** 10.3390/ijms14011516

**Published:** 2013-01-14

**Authors:** Muhammad Asif Arif, Wolfgang Frank, Basel Khraiwesh

**Affiliations:** 1 Department of Molecular Developmental Biology, Nijmegen Center for Molecular Life Sciences, 6500 HB Nijmegen, The Netherlands; E-Mail: masifarif@gmail.com; 2 Plant Molecular Cell Biology, Department I, Faculty of Biology, Ludwig-Maximilians-University Munich, LMU Biocenter, Grosshaderner Strasse 2-4, 82152 Planegg-Martinsried, Germany; E-Mail: wolfgang.frank@lmu.de; 3 Center for Desert Agriculture, Division of Biological and Environmental Sciences and Engineering, King Abdullah University of Science and Technology, Thuwal 23955-6900, Kingdom of Saudi Arabia

**Keywords:** RNAi, non-coding RNAs, miRNA, siRNA, gene silencing, *Physcomitrella patens*

## Abstract

RNA interference (RNAi) is a mechanism that regulates genes by either transcriptional (TGS) or posttranscriptional gene silencing (PTGS), required for genome maintenance and proper development of an organism. Small non-coding RNAs are the key players in RNAi and have been intensively studied in eukaryotes. In plants, several classes of small RNAs with specific sizes and dedicated functions have evolved. The major classes of small RNAs include microRNAs (miRNAs) and small interfering RNAs (siRNAs), which differ in their biogenesis. miRNAs are synthesized from a short hairpin structure while siRNAs are derived from long double-stranded RNAs (dsRNA). Both miRNA and siRNAs control the expression of cognate target RNAs by binding to reverse complementary sequences mediating cleavage or translational inhibition of the target RNA. They also act on the DNA and cause epigenetic changes such as DNA methylation and histone modifications. In the last years, the analysis of plant RNAi pathways was extended to the bryophyte *Physcomitrella patens*, a non-flowering, non-vascular ancient land plant that diverged from the lineage of seed plants approximately 450 million years ago. Based on a number of characteristic features and its phylogenetic key position in land plant evolution *P. patens* emerged as a plant model species to address basic as well as applied topics in plant biology. Here we summarize the current knowledge on the role of RNAi in *P. patens* that shows functional overlap with RNAi pathways from seed plants, and also unique features specific to this species.

## 1. Introduction

Small non-coding RNAs have been increasingly investigated as important regulators of gene expression. These small RNAs of 20–24 nucleotides (nt) function by causing either TGS or PTGS [1–5]. They were first discovered in the nematode *Caenorhabditis elegans* [6] and are responsible for the phenomenon known as RNAi, co-suppression, gene silencing, or quelling [7–10]. Shortly after these reports were published, it was shown that PTGS in plants is correlated with the activity of small RNAs [11]. These small RNAs regulate various biological processes in animals and plants by interfering with mRNA translation or directing target RNA cleavage, which is the predominant mode of action in plants. In plants, several classes of small RNAs with specific sizes and dedicated functions have evolved through a series of pathways, namely miRNAs, repeat-associated small interfering RNAs (ra-siRNAs), natural antisense transcript-derived small interfering RNAs (nat-siRNAs), and trans-acting small interfering RNAs (ta-siRNAs) [12–16].

In recent years, the analysis of plant RNAi pathway has been extended to the bryophyte (moss) *P. patens*, a non-vascular and a non-flowering ancient land plant, that diverged from the higher plants approximately 450 million years ago [17]. *P. patens* occupies an important phylogenetic position to study the development of higher plants and the adaptation to the land environment. In terms of evolutionary distance of *P. patens* to flowering plants, it equals the evolutionary distance from fish to humans. *P. patens* has emerged as a model plant species to address basic as well as applied topics in plant biology. *P. patens* exhibits a high frequency of homologous recombination which makes it an ideal model system for reverse genetics approaches by the simple generation of targeted gene knockout mutants [18]. Furthermore, the *P. patens* genome is available that makes it a valuable tool for reconstructing the evolution of plant genomes and functional genomics approaches [19].

Recently, a small RNA database has been established in *P. patens* [5,12,20–23]. Besides the analysis of *P. patens* small RNA pathways, molecular tools were developed exploiting the mode of action of small RNAs for the down-regulation of genes in reverse genetics applications. These approaches include the use of conventional inverted RNAi constructs [24,25] as well as the expression of highly specific artificial miRNAs [26]. Even though the major RNAi pathways are evolutionarily conserved in *P. patens*, there are particular differences in the functional components of small RNA pathways and the biological function of small RNAs. These include a specific amplification of initial miRNA and ta-siRNA signals by the generation of transitive siRNAs, deviating functions and specificities of DICER-LIKE proteins and an epigenetic gene silencing pathway that is triggered by miRNAs. These findings underline that *P. patens* serves as a valuable model system to study the evolution, diversity, and complexity of plant RNAi pathways.

## 2. *Physcomitrella patens* Small RNAs

**Figure 1:**
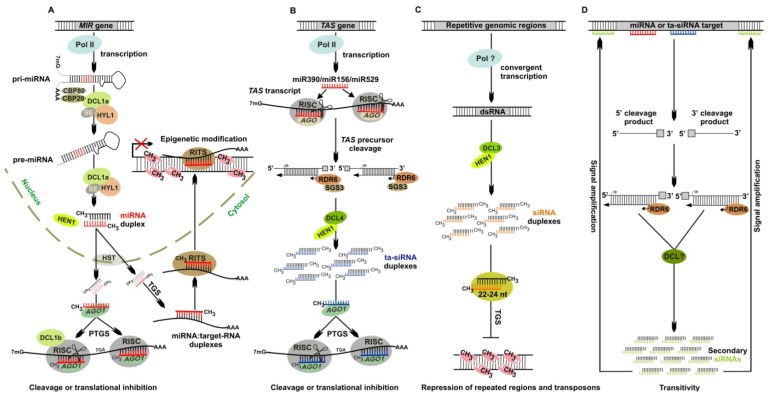
Different endogenous small interfering RNA (siRNA) pathways of *P. patens*. Only PpDCLs and PpRDR6 have been functionally characterized in *P. patens*; evidence for proteins shown in figure comes from *Arabidopsis* and their homologous exist in *P. patens*. (**A**) *P. patens* miRNA pathway. *MIR* genes are transcribed by RNA polymerase II into pri-miRNA transcripts that are further processed into pre-miRNAs harboring a characteristic hairpin structure. From the stem of the pre-miRNA the miRNA/miRNA* duplex is excised by PpDCL1a and can be assisted by HYL and SE proteins. These are then methylated by HUA ENHANCER 1 (HEN1) and transported to the cytoplasm by HASTY (HST). The miRNA guide strand is selected, incorporated, and stabilized in dedicated AGO1 protein. miRNA-guided AGO1-containing RNA-induced silencing complex (RISC) directs mRNA cleavage or translation inhibition of the target transcript. Highly abundant miRNAs are either loaded into a RITS complex and subsequently interact with their target to form a duplex, or these duplexes are formed at first and then loaded into RITS. The miRNA:RNA duplexes bound by RNAi-induced transcriptional silencing complex (RITS) initiate DNA methylation at complementary genomic loci. (**B**) *P. patens* ta-siRNA pathway. *TAS* genes are transcribed by RNA polymerase II into *TAS* precursors harbouring miR390, miR156 and miR529 binding sites. After *TAS* precursor cleavage at these miRNA sites the middle cleavage product is converted into double-stranded RNAs (dsRNA) by PpRDR6 and subsequently processed into phased ta-siRNAs by PpDCL4. ta-siRNAs are loaded into RISC where they act like miRNAs. (**C**) *P. patens* siRNA pathway from repetitive genomic regions primarily LTR-retrotransposons and helitron DNA transposons. dsRNA processed into siRNAs by PpDCL3 and HEN1-mediated siRNA stabilization, the PpDCL3-dependent 22–24 nt siRNAs caused a de-repression of LTR retrotransposon-associated reverse transcriptases pointing to an epigenetic control of these elements. (**D**) Secondary siRNAs in *P. patens*. dsRNA is synthesised from cleaved miRNA or ta-siRNA targets by RdRP and processed into secondary siRNAs that mediate cleavage of the target RNA upstream and downstream of the miRNA/ta-siRNA recognition motif resulting in an amplification of the initial small RNA trigger.

High throughput sequencing approaches led to the identification of small RNAs from diverse plant species with specific origins, sizes and functions [2]. Several classes of small RNA have been identified in *P. patens* which can be distinguished based on their specific biogenesis: miRNAs, ta-siRNAs, ra-siRNAs and secondary siRNAs (Figure 1A–D) [2,20,21,23,27]. In general, small RNAs are generated from complete or partially dsRNA precursors by the action of DICER-LIKE (DCL) proteins [1,28]. The small RNA duplexes generated by Dicer activity have a characteristic 2-nucleotide overhang at the 3′ end due to an offset slicing activity of the DCL proteins. In plants these 3′ overhangs are stabilized by 2′-*O*-methylation [29–32]. Only one strand of the processed small RNA duplex subsequently associates with an RNA-induced silencing complex (RISC) that scans for nucleic acids complementary to the loaded small RNA to execute its function [33–36]. In plants, small RNAs act in gene silencing by different ways, namely by mediating RNA slicing [37–40], translational repression [41–43], and histone modification and DNA methylation [5,44,45]. The first two mechanisms control gene expression posttranscriptionally, whereas the latter affects gene expression at the transcriptional level.

### 2.1. miRNAs (microRNAs)

miRNAs are small RNAs of 20–22 nt that are encoded by endogenous *MIR* genes. In the miRNA biogenesis pathway, primary miRNAs (pri-miRNAs) are transcribed from *MIR* genes by RNA polymerase II (Pol II) into transcripts harboring a characteristic hairpin [46] (Figure 1A). In *Arabidopsis* processing of pri-miRNAs into pre-miRNAs is catalyzed by DCL1 and assisted by HYPONASTIC LEAVES 1 (HYL1) and SERRATE (SE) [36]. The pre-miRNA hairpin precursor is further processed into a 20–22 nt miRNA/miRNA* duplex by DCL1. This duplex is then methylated at the 3′ terminus by HUA ENHANCER 1 (HEN1) and exported into the cytoplasm by HASTY (HST) protein [47,48]. Within the cytoplasm, one strand of the duplex (the miRNA) is incorporated into RISC and guides binding of the complex to cognate target transcripts by sequence complementarity. In *P. patens PpDCL1a* is responsible for production of miRNAs [5]. In addition to the control of targets at the posttranscriptional level, *P. patens* miRNAs regulate gene expression by causing epigenetic changes such as DNA methylation (Figure 1A) [5,49–51].

**Figure 2:**
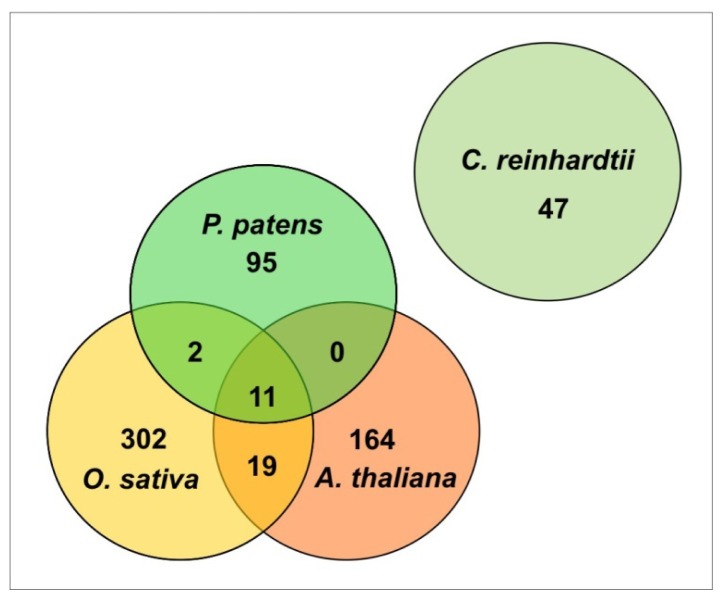
Venn diagram comparing miRNA families from the seed plants *A. thaliana* (dicot) and *O. sativa* (monocot), the moss *P. patens* and the unicellular alga *C. reinhardtii* based on miRBase database (Release 19.0, http://www.mirbase.org/). *C. reinhardtii* miRNA families are species-specific since they do not show sequence similarity to miRNA families from land plants. 11 miRNA families (miR156, miR160, miR166, miR167, miR171, miR319, miR390, miR395, miR408, miR414 and miR419) are conserved between *A. thaliana*, *O. sativa*, and *P. patens.* Two additional miRNA families, (miR529 and miR535) are conserved between *O. sativa* and *P. patens*.

Independent studies on small RNAs in *P. patens* revealed the existence of a diverse miRNA repertoire including highly conserved miRNA families [2,12,20,23,27]. Conserved miRNAs families between *P. patens* and other land plants were discovered using the microHARVESTER algorithm that analyszes and predicts conserved *MIR* genes in genomic sequences [20]. The reported *P. patens* miRNAs were identified from wild type plants covering major developmental stages (protonema, young gametophores, gametophores, and sporophytes). Further, the identification of miRNAs was restricted to plants that were cultivated under standard growth conditions, and thus miRNAs which respond to certain physiological conditions such as abiotic stress may have escaped identification. These analyses led to the identification of 108 miRNA families in *P. patens*. Interestingly, 11 miRNA families are conserved between *P. patens*, *A. thaliana* and *O. sativa*, while the 47 miRNAs families identified in the green alga *C. reinhardtii* are species-specific and are not related to land plant miRNAs (Figure 2).

Certain miRNAs regulate various key developmental programs including auxin signaling, lateral root formation, leaf development and polarity, meristem boundary formation and organ separation, transition from the juvenile-to-adult vegetative phase and vegetative-to-flowering phase, floral organ identity, and reproduction, and also plant responses to different stress conditions [50]. Most conserved miRNAs target mRNAs of conserved transcription factor families, for example miR156, miR159/319, miR160, miR166 andmiR171 target transcripts encoding SBPs, MYBs/TCPs, ARFs, HD-ZIPs andGRAS domain proteins, respectively [50]. Some conserved miRNAs and their targets have been identified in *P. patens*, but functional analysis in *P. patens* is still required to address this interesting topic of the evolution of miRNAs in plants. Some moss-specific miRNAs that target mRNAs encoding transcription factors that are associated with developmental control have been functionally characterized. For example, miR534 controls mRNAs encoding ankyrin repeat containing proteins homologous to the *A. thaliana BLADE ON PETIOLE 1* and *2*, miR538 cleaves mRNAs of the *MADS* box transcription factor family and miR902 controls several mRNAs coding for basic helix-loop-helix transcription factors [2,27,52]. Some miRNAs are expressed in a restricted spatiotemporal manner, so they control target genes only in specific expression domains to establish boundaries for developmental decisions. One example is the cell-type specific and cytokinin-dependent repression of the *P. patens* specific miR534a that acts in the juvenile-to-adult transition and controls bud formation through an accompanying derepression of *PpBOP* expression within the same cells [53].

### 2.2. ta-siRNAs (Trans-Acting Small Interfering RNAs)

Ta-siRNAs are plant specific small RNAs that are generated from genome encoded *TAS* transcripts; initially the *TAS* precursor is cleaved by a miRNA, subsequently converted into double stranded RNA and processed into phased siRNA duplexes by DCL proteins (Figure 1B). In *A. thaliana* main components of ta-siRNA pathway are DCL4, RDR6, SGS3, and DRB4 proteins [13,14,16,54–57]. In *A. thaliana* four *TAS* gene families (*TAS1-4*) have been identified. miR173-mediated cleavage generates ta-siRNA from *TAS1* and *TAS2* precursors, miR390 is required for *TAS3* cleavage while miR828 is assigned to *TAS4. TAS2* and *TAS4* are encoded by single genes while *TAS1* and *TAS3* each harbour three members [13–16,57]. In *A. thaliana* most miRNAs are incorporated into ARGONAUTE1 (AGO1)-containing RISC to direct cleavage of their targets, whereas miR390 specifically interacts with AGO7. The miR390-AGO7 complex has a specific role in the phased processing of ta-siRNAs from *TAS3* precursors [58]. Like *A. thaliana* the rice genome also encodes three *TAS3* precursors (*TAS3a-c*) and all *TAS3* precursors from both species have dual miR390 binding sites but miR390-directed cleavage only occurs at the 3′ miR390 binding site. Consequently, ta-siRNAs are only generated from the cleavage product located 5′ to the cleaved miR390 site [4,23]. Recently a new *TAS* family, *TAS5*, has been identified in tomato, which required miR482-directed initial slicing [59]. The *P. patens* genome encodes *TAS3* and *TAS6* families. The *P. patens TAS3* family comprises six members (*TAS3a–f*) each harbouring dual miR390 sites. The *TAS3* precursors are cleaved at both miR390 binding sites and subsequently the middle cleavage product is converted into dsRNA by PpRDR6 (Figure 1B) [23]. The *P. patens TAS6* family is encoded by three loci (*TAS6a–c*) that are in close proximity to *TAS3* loci and are typified by sliced miR156 and miR529 target sites [60]. *P. patens* Δ*PpDCL1a*, Δ*PpRDR6* and Δ*PpDCL4* mutants lack ta-siRNA production based on specific functions of the respective proteins in ta-siRNA biogenesis: the absence of ta-siRNAs in the Δ*PpDCL1a* mutant is due to the lack of the specific miRNAs (miR390, miR156 and miR529) that are required to initiate the ta-siRNA pathway by the cleavage of the respective *TAS* precursors, *PpRDR6* the essential enzyme catalysing the conversion of cleaved precursors into dsRNA while *PpDCL4* is the DCL protein that processes ta-siRNAs from the dsRNA precursors in a phased manner [5,12,60].

Like for miRNAs, ta-siRNAs targets can be predicted on the basis of sequence complementarity between a ta-siRNA and its target RNA. In *A. thaliana TAS3* ta-siRNAs regulate several *AUXIN RESPONSE FACTOR* (*ARF*) mRNAs including *ARF3* and *ARF4* which regulate leaf polarity and proper timing of vegetative shoot development [13,54,56,61–63]. Likewise, rice *TAS3*-derived ta-siRNAs control *ARF2/3* [4]. Strikingly, *P. patens TAS3* ta-siRNAs, also target *ARF* transcripts (Phypa_203442, Phypa_224167) even though the sequences of the respective ta-siRNAs are not conserved between moss and seed plants [2]. These observations indicate that at least *ARF* targeting *TAS3* ta-siRNA function is conserved between *A. thaliana*, rice and *P. patens* irrespective of the varying ta-siRNA sequences which indicate a strong evolutionary conservation of *TAS3* ta-siRNAs and *ARF* regulation in mosses and seed plants.

Besides *ARF* transcripts, *P. patens TAS3* ta-siRNAs also regulate an mRNA encoding an AP2/EREPB transcription factor (Phypa_65352) [12]. *AtTAS1* and *AtTAS2* family ta-siRNAs target PPR (pentatricopeptide repeat) transcripts whereas *MYB* transcription factor transcripts are regulated by *AtTAS4* ta-siRNAs [13–16]. Tomato *TAS5* ta-siRNA target the resistance gene *Bs4*, and *P. patens TAS6* ta-siRNA targets an mRNA encoding a zinc finger protein (Pp1s286_43v6.1) [59,60]. In addition to their role in mediating RNA target cleavage *A. thaliana* ta-siRNAs may also function in the nucleus to control mRNA splicing. A binding site of a *TAS1a*-derived ta-siRNA was found in an intron of a pre-mRNA that encodes a FAD binding domain-containing protein (*At2g46740*) and elevated levels of unspliced *At2g46740* mRNA was detected in ta-siRNA deficient mutants [16]. However, evidence for a role of *P. patens* ta-siRNAs in the control of RNA splicing is still missing.

### 2.3. ra-siRNAs (Repeat Associated Small Interfering RNAs)

dsRNAs are sources of siRNAs biogenesis and they can have different origins such as RNA transcribed from inverted repeats, pairing of natural *cis*-antisense transcripts, transcript from retroelement-rich genome regions, synthesis by the action of RNA-dependent RNA polymerases (RDRs) or the replication of RNA viruses [64,65]. dsRNAs are cleaved into 21–24 nt siRNAs by DCL proteins whereby the size of the siRNAs depends on the specific DCL protein that catalyzes the processing of siRNAs from these precursors (Figure 1C). Like miRNAs, siRNAs are loaded into AGO protein containing RISC that guide recognition of target genes by base pairing and silence them [39,66]. In addition, siRNAs can be incorporated into RNAi-induced transcriptional silencing complex (RITS) and transcriptionally silence the loci mainly by DNA methylation. For most of the ra-siRNAs this is the predominant mode of action in particular for all those that are derived from repetitive genomic regions. The first proof of siRNA generation and subsequent gene silencing in *P. patens* was obtained from the expression of inverted *GUS* and *GFP* RNAi constructs that caused silencing of GUS and GFP signals in *P. patens* lines constitutively expressing *GUS* and *GFP* [24,25]. The total endogenous small RNA population of flowering plants is characterized by two distinct peaks at 21 nt and 24 nt [15,67,68]. The 21nt size constitutes mainly miRNAs and ta-siRNAs while 24 nt small RNAs are predominantly generated from intergenic and repetitive genomic regions [15,68]. In *A. thaliana* 24 nt small RNAs are primarily generated by AtDCL3 [69].

The *P. patens* top 100 non-miRNA and non-ta-siRNA siRNA producing regions fall into two distinct categories and were classified on the basis of generated siRNA size and abundance [21]. One class is dominated by 21 nt RNAs whereas the second class comprises a mixture of 21–24 nt RNAs in a strikingly consistent ratio. The loci generating these two types of small RNAs were annotated as *Pp21SR* (21 nt small RNA) and *Pp23SR* (21, 23, and 24 nt small RNA) loci, respectively [21]. The *Pp23SR* loci constitute larger genomic regions between 5 and 50 kb in length (median 11.9 kb) whereas the *Pp21SR* loci cover small regions that range from 100 to 1000 nt in length (median 247.5 nt). *Pp21SR* loci mainly generate single stranded small RNA precursors for siRNA production while *Pp23SR* loci are templates for the production of long dsRNA precursors which are processed into siRNAs as inferred from the sense and antisense polarity of siRNAs derived from these loci. Most of the *Pp23SR* loci overlap with LTR-retrotransposons and helitron elements, and PpDCL3 generates 22–24 nt siRNA from these loci [21]. *Pp23SR* loci are characterized by dense cytosine-methylation and the depletion of the 22–24 nt siRNAs that originate from these loci in Δ*PpDCL3* mutants caused a de-repression of LTR retrotransposon-associated reverse transcriptases indicating an epigenetic control of these elements by the specific set of 22–24 nt siRNAs (Figure 1C). Thus, the 22–24 nt small RNAs from *Pp23SR* loci are functionally similar to AtDCL3 generated 24 nt siRNAs and are involved in the repression of transposons [21]. The biogenesis pathways and functions of siRNAs that derive from *Pp21SR* loci and of the 21 nt siRNA fraction originating from *Pp23SR* loci are not clear yet.

### 2.4. Secondary siRNAs

Recent studies involving plants and the nematode *C. elegans* reported the amplification of silencing related RNA and explain how strong, persistent silencing can be initiated with small amounts of initiator dsRNA [23,70,71]. The amplification process has implications for application of RNAi to control gene expression in biotechnology and for understanding the effects of silencing RNAs on cell function and organism development. The initiator of transitivity is a dsRNA that is first processed by Dicer into siRNA or a related type of RNA referred to as miRNA, these 21–25 nucleotide single stranded RNAs are the primary silencing RNAs in the transitive process [72,73]. These siRNAs can not only trigger their cleavage and subsequent degradation of target RNAs, but also serve as primers for RdRP activity. With the help of RdRP target RNAs are converted into double strands RNAs which serves as templates for the production of secondary siRNAs by Dicer action, in this way siRNA population is generated which is distinct from the initiator siRNA [73,74]. In plants, the transitivity occurs in both directions of the initial siRNA trigger whereas in animals spreading of the initial signal occurs only upstream of the trigger [23,73–78].

In *P. patens* amplification of miRNA-mediated RNA cleavage sites by 5′RACE yielded several products including the expected miRNA-directed RNA cleavage products. These additional degradation products are the consequence of the action of secondary siRNAs that guide cleavage of the miRNA targets at additional sites [5]. These secondary siRNAs biogenesis involves RDR6 activity and secondary siRNAs were detected both in sense and antisense orientation [5]. Furthermore, secondary siRNAs were generated from upstream and downstream regions relative to the miRNA binding site (Figure 1D) [5]. Secondary siRNAs were also generated after ta-siRNA-mediated cleavage of the ta-siRNA target *PpEREBP/AP2*. The biogenesis of transitive siRNAs in *P. patens* is different from biogenesis of secondary siRNAs in *C. elegans* where transitivity only occurs in antisense polarity due to an unprimed *de novo* synthesis of dsRNA by RdRP [70,71]. Furthermore, transitivity in *P. patens* only occurs after miRNA or ta-siRNA mediated cleavage of target RNAs, since Δ*PpDCL1b* mutants defective in target cleavage do not generate transitive siRNAs [5]. Recently it is shown that secondary siRNA biogenesis in *A. thaliana* is triggered by 22 nt rather than by the more typical 21 nt miRNAs and ta-siRNAs. So for eight *A. thaliana* miRNAs (miR168, miR173, miR393, miR447, miR472, miR473, miR828 and miR856) and one ta-siRNA (ta-siR2140) are known triggers of siRNA production [79]. Since secondary siRNAs in *P. patens* are generated from RNAs that are targeted by 21nt miRNAs (miR160, miR166) and a 21nt ta-siRNA (ta-siRNA 6(+)) further studies are required to address the differences and specificities in secondary siRNA production in mosses and seed plants.

## 3. *Physcomitrella patens* Homologues of RNAi Pathway Components

In *P. patens* the functional analysis of RNAi pathway components is limited to *PpDCL1a*, *PpDCL1b*, *PpDCL3*, *PpDCL4*, and *PpRDR6.* To obtain a comprehensive view of the genes that are present in *P. patens*, we used *A. thaliana* proteins that are involved in different small RNA pathways as queries for BLASTP searches in *P. patens* V1.6 protein database (available online: http://www.cosmoss.org (accessed on 10 December 2012)). This *in silico* analysis identified presence of *A. thaliana* homologues of all known protein families involved in small RNA pathways in *P. patens* (Table 1) that indicate a wide conservation over evolutionary time and point to large functional overlaps in different plant species. However, the size of certain RNAi protein families varies between *P. patens* and seed plants. For example, six AGO family members were found in *P. patens* whereas ten members are present in *A. thaliana* [2,37]. These ten AGO proteins are clustered into three clades: first clade comprises AtAGO1, AtAGO5 and AtAGO10, second clade has AtAGO2, AtAGO3 and AtAGO7, and third clade includes AtAGO4, AtAGO6, AtAGO8 and AtAGO9 proteins [80]. *P. patens* encodes three homologues of AtAGO1 which is the core component of RISC, and three homologous of AtAGO4, AtAGO6 and AtAGO9 proteins, respectively (Table 1). Thus, beside a large overlap of small RNA-related proteins, there are particular differences in the protein repertoire that may cause deviating functions of small RNA pathways in *P. patens* and seed plants.

**Figure 3:**
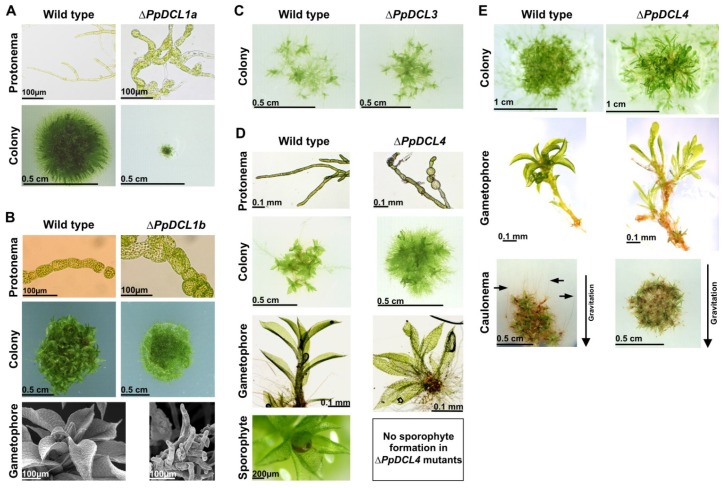
*P. patens DCL* mutant phenotypes. (**A**) Phenotypic comparison of *P. patens* wild type and a Δ*PpDCL1a* mutant line: in mutants cell size and shape is affected, they have retarded growth, and developmental arrest at the filamentous protonema stage is obserbed. (**B**) Phenotypic comparison of *P. patens* wild type and a Δ*PpDCL1b* mutant line: mutants show deviating cell division, cell size, cell shape and growth polarity, and they developed only a small number of gametophores which in addition are malformed (**C**) Phenotypic comparison of *P. patens* wild type and a Δ*PpDCL3* mutant line: mutants show accelerated gametophore development. (**D**) Phenotypic comparison of *P. patens* wild type and Δ*PpDCL4* mutant lines under standard growth conditions: mutants show several brachycytes in the protonema, colony produces relative more protonema and less leafy gametophores, gametophores are stunted in growth, and mutants are sterile (**E**) Phenotypic comparison of *P. patens* wild type and Δ*PpDCL4* mutant lines under short day conditions: mutants generates malformed gametophores, and fail to produce caulonema under dark conditions.

Like *A. thaliana*, there are four DCL proteins present in *P. patens* (Table 1) [2], but the DCL repertoire differs. *P. patens* encodes two proteins similar to AtDCL1 and two DCL proteins homologous to AtDCL3 and AtDCL4 respectively, whereas no AtDCL2 homologue is present in *P. patens* (Table 1). Consequently, the *P. patens* proteins were named PpDCL1a, PpDCL1b, PpDCL3 and PpDCL4. Although PpDCL1a and PpDCL1b are highly similar to the *A. thaliana* AtDCL1 protein but experimental evidences indicate distinct functions [5]. Strongly reduced expression levels or complete lack of miRNAs and elevated steady-state transcript levels of cognate miRNA targets were detected in Δ*PpDCL1a* null mutants [5]. This is similar to *A. thaliana* where *dcl1* mutants that lack miRNAs accompanied with increased miRNA target expression levels in addition, *Atdcl1* mutants are embryo lethal [29,134]. Similarly, Δ*PpDCL1a* mutants displayed severe developmental disorders affecting cell size and shape, retarded growth that was partially complemented by growth on medium supplemented with vitamins, and developmental arrest at the filamentous protonema stage since these mutants failed to developed leafy gametophores (Figure 3A). The lack of gametophores also causes sterility of Δ*PpDCL1a* mutants since the gametophores bear the male and female sex organs (antheridia and archegonia). So, it was concluded that PpDCL1a is the functional equivalent to the AtDCL1 protein from *A. thaliana*. Similar to Δ*PpDCL1a* mutants, Δ*PpDCL1b* mutants showed developmental disorders (Figure 3B) including abnormalities in cell division, cell size, cell shape and growth polarity, and they developed only a small number of gametophores which in addition were malformed [5]. PpDCL1b is involved in miRNA directed target genes cleavage, this is a novel mechanism not known so far in other organism.

The *P. patens* PpDCL3 generates 22–24 nt small RNAs mainly from intergenic regions repetitive regions. Repetitive siRNAs control *P. patens* development since Δ*PpDCL3* mutants show an accelerated gametophore formation (Figure 3C) [21]. PpDCL3 generated 22–24 nt are analogous in function to AtDCL3 generated 24 nt siRNAs and are involved in the repression of transposons [21]. The Δ*PpDCL4* loss-of-function mutants have dramatic developmental abnormalities [60], mutants display phenotypic aberration throughout all the developmental stages and in addition are sterile (Figure 3D,E). In *Arabidopsis dcl4* mutants, the lack of ta-siRNAs only has minor developmental effects, namely the formation of slightly elongated and down-ward curled rosette leaf margins and accelerated juvenile-to-adult vegetative phase changes [54,55]. *P. patens* Δ*PpRDR6* mutants show mild phenotypic deviation, only an accelerated transition from juvenile to adult gametophyte stage was observed [12]. Similarly *A. thaliana rdr6* mutants exhibit mild phenotypic deviations, rosette leaves are elongated and slightly downwards curled, and an accelerated transition to the adult phase [14]. Like *Arabidopsis* and *P. patens* rice, *rdr6* (*OsSHL2*) is required for ta-siRNA production but in contrast, strong *rdr6* mutants display severe phenotypes and they lack the shoot apical meristem [135,136] whereas the weak mutations leads to defects in the adaxial-abaxial patterning of floral organs [137]. These severe phenotypes of rice hint to a broader activity of rdr6 protein, it could be also an indication of the stronger ta-siRNA directed regulation on developmental programs of rice.

## 4. *Physcomitrella patens* and Epigenetic Modification

In some cases, endogenous siRNAs trigger epigenetic effects at target loci and are associated with RNA-directed DNA methylation (RdDM) and chromatin remodeling [69,92,138]. In plants, dsRNAs which contain sequences that are homologous to promoter regions can trigger promoter methylation and transcriptional gene silencing [139,140]. RdDM leads to *de novo* methylation of cytosine that occurs in all sequence contexts, not just in symmetrical CG dinucleotides, which are considered the usual targets for methylation. Methylation is largely restricted to the region of RNA-DNA sequence homology [141,142]. So, unlike RNAi based heterochromatin, which can spread over several kilobases from the RNA-targeted nucleation site, RdDM does not usually infiltrate substantially into adjacent sequences [143]. RdDM is a stepwise process that is initiated by RNA signals and site-specific DNA methyltransferases [121,144]. The methylation of cytosine residues can result in transcriptional silencing. Recently in *Arabidopsis thaliana* a nucleolar complex involved in the siRNA-directed silencing of endogenous repeat regions has been identified. This complex constitutes several proteins which are linked to RdDM including RDR2, DCL3 and AGO4. By the action of this nucleolar complex 24 nt siRNAs generated from repetitive regions methylate DNA and do a role in silencing of repetitive regions. Homologues of silencing nucleolar complex are also present in *P. patens* (Table 1), functional analyses is required to obtain further mechanistic insight into *P. patens* RdDM pathways.

In yeast *Schizosaccharomyces pombe*, an RITS contains AGO1, a chromodomain protein Chp1, and TAS3 [145]. RITS and Rdrp1 are recruited to silenced loci [102] and it is proposed that it is involved in a self-reinforcing loop, in which DNA-interacting proteins and the siRNAs loaded RITS guide methylation of lysine 9 or histone 3 that result in heterochromatin formation. If a transcript is formed from these loci it will be converted to siRNA precursors by Rdrp1 and will keep loci in heterochromatin state [34]. However, it is not clear whether siRNAs alone target the complex directly to the genomic DNA or if the interaction requires an additional RNA molecule closely associated with the genomic DNA.

In *P. patens* PpDCL3-dependent 22–24 nt siRNAs are involved in epigenetic silencing of LTR retrotransposons [21]. Additional evidence for the existence of small RNA-mediated epigenetic gene silencing was obtained from the analysis of Δ*PpDCL1b* mutants [5]. In Δ*PpDCL1b* mutants miRNA-triggered cleavage of target RNAs was abolished, but it is unlikely that PpDCL1b is directly involved in the cleavage of target RNAs since AGO proteins residing in RISC are the catalytic enzymes responsible for RNA-directed target cleavage [66]. Thus, similar to animal dicers which were shown to be components of RISC-loading complexes (RLC) PpDCL1b was proposed to play a role in loading miRNAs into RISC [66,146–148] (Figure 1A). Even though miRNA-directed cleavage of target RNAs was abolished in the Δ*PpDCL1b* mutants and elevated steady-state transcript levels of miRNA targets were expected, all analyzed miRNA targets had strongly reduced expression levels in Δ*PpDCL1b* mutants. Subsequent analysis of revealed cytosine methylation at CpG residues within the cognate miRNA target loci causing transcriptional silencing of these loci. Furthermore, DNA methylation was not restricted to the region of the encoded miRNA binding site, but spread into upstream and downstream regions including introns and promoter regions [5]. In two miRNA target genes, *PpHB10* and *PpC3HDZIP1*, the miRNA binding site is disrupted by intron making it unlikely that DNA methlyation is initiated by the formation of an miRNA:DNA hybrid. In addition, stable miRNA:mRNA duplexes were observed in the Δ*PpDCL1b* mutants leading to the hypothesis that these duplexes interact with RITS to guide it to cognate genomic regions to initiate DNA methylation (Figure 1A). Moreover, it was hypothesized that DNA methylation of miRNA target genes in the Δ*PpDCL1b* mutants is triggered by a high miRNA:target RNA ratio due to the abolished target cleavage [5]. The influence of the miRNA:target RNA ratio on DNA methylation and transcriptional silencing was also proved by the analysis of miR1026 and its target gene *PpbHLH* in *P. patens* wild type. Upon ABA treatment miR1026 accumulates to elevated steady-state levels which results in a higher miR1026:*PpbHLH.* Consequently, reduced *PpbHLH* steady-state levels were observed which, at least in part, are caused by transcriptional silencing of the *PpbHLH* locus due to DNA methylation at CpG sites within the *PpbHLH* genomic locus.

## 5. *Physcomitrella patens* and Autoregulation of miRNA Biogenesis

The miRNA pathway controls many important genes, and hence requires proper regulation of its own pathway. One of the regulations might be regulation of transcripts encoding catalytic small RNA pathway components and indeed miRNAs are involved in the negative feedback control of important miRNA pathway genes. For example, in *A. thaliana* miR162 targets the *AtDCL1* transcript which is the key gene of miRNA biogenesis [149]. Another feedback control may affect the maturation of the *AtDCL1* pre-mRNA. Intron 14 of the *AtDCL1* gene harbours a miR828 precursor sequence [15]. Processing of the miR828 by AtDCL1 could compete with the splicing of the *AtDCL1* pre-mRNA to control functional *AtDCL1* mRNA levels. So far, no miRNA has been identified that targets *PpDCL1a* or *PpDCL1b*. However, miR1047 precursor is present in intron 7 of *PpDCL1a* that is essential for miRNA biogenesis, suggesting a similar role of miR828 in *AtDCL1* [2]. Another conserved miRNA-mediated feedback was reported for *AGO1* mRNAs in *A. thaliana* and *P. patens*, AGO1 is a key protein in RISC and is required for miRNA directed cleavage of target transcripts [89,150]. In *A. thaliana* the single *AtAGO1* mRNA is targeted by miR168, whereas miR904 targets three *PpAGO1* homologues (*PpAGO1a–c*) [2,88,89]. This present a negative feedback loop, perturbation of this control loop by the expression of a miR168-resistant *AtAGO1* mRNA led to elevated *AtAGO1* transcript levels and an increased abundance of miRNA target RNAs suggesting that elevated AtAGO1 levels interfere with proper miRNA-RISC activity. In addition, miR168-resistant *AtAGO1* lines show developmental defects indicating the biological relevance of this negative control loop [89]. Further studies are required to know whether the miR904-mediated control of the *P. patens PpAGO1a–c* homologues has a similar function in the maintenance of miRNA-RISC activity. In addition, functional analysis will reveal if the three *PpAGO1* homologues act redundantly and are functionally equivalent to the single *A. thaliana AtAGO1* or they exhibit diverse functions. The intronic mature miRNAs or its precursor sequences of ath-miR838 and ppt-miR1047 regulating DCL1 or and ath-miR168 and ppt-miR904 targeting *AGO1* transcripts are not conserved between both species pointing to a convergent evolution of these control pathways.

## 6. *Physcomitrella patens* and Artificial miRNAs

The successful use of artificial miRNAs (amiRNAs) for the specific down-regulation of genes was shown for the dicotyledonous plants *Arabidopsis*, tomato and tobacco, and for the monocot rice [150–156]. In most of the cases the amiRNA was expressed from endogenous miRNA precursors. However, high expression rates of amiRNAs were achieved in tobacco and tomato using the *Arabidopsis* miR164b precursor sequence indicating correct processing of conserved pre-miRNAs within seed plants [150]. The *Arabidopsis* miR319a precursor was used for the expression of amiRNAs in *P. patens* targeting *PpFtsZ2-1* and *PpGNT1* genes [26]. The constitutive expression of the modified precursor for both amiRNAs resulted in precisely processing of mature miRNA and effective highly specific knockdown of the corresponding transcripts. The amiRNA-mediated knockdown of *PpFtsZ2-1* that is indispensable for plastid division caused the formation of macrochloroplasts which is the exact phenotypic deviation observed in Δ*PpFtsZ2-1* null mutants [157]. In addition, similar to natural *P. patens* miRNAs the overexpression of amiRNAs caused transitivity by the generation of secondary siRNAs [26]. The expression of amiRNAs can be beneficial to target several related genes or they can be expressed by tissue specific or inducible manner. The use of amiRNA may replace conventional inverted repeat-based RNAi constructs because of off-targeting effects [25].

## 7. Conclusions and Future Prospects

Understanding of *P. patens* RNAi pathways was made by high-throughput and “degradome” sequencing as well as the functional analysis of essential components of RNAi. In future, a combination of these techniques and the inclusion of gene expression profiling using RNA-seq technique can be applied to the available *P. patens* mutants with perturbed small RNA pathways. The information obtained from such analyses will add to a comprehensive understanding of small RNA pathways on a genome-wide scale.

The functional analysis of RNAi components in *P. patens* is currently limited to *ΔPpDCL1a*, *ΔPpDCL1b*, *ΔPpDCL3*, *ΔPpDCL4 and ΔRDR6* mutants. However, it is now evident that *P. patens* RNAi pathway have specific features that differ from seed plants. Since all identified components of seed plant RNAi pathway have homologues in *P. patens*, their detailed analysis may reveal further conserved or deviating functions. In addition, functional studies of *P. patens* miRNAs by miRNA overexpression, miRNA target mimicry or the generation of miRNA-resistant lines by altering miRNA binding sites may reveal interesting findings. The analysis of conserved miRNAs together with their conserved targets between seed plants and *P. patens* will clarify whether these miRNAs control homologous and/or analogous processes.

The adaptation to adverse environmental conditions is a major prerequisite to assure survival of plants in nature, analyzing the involvement of small RNAs in the control of stress-induced gene regulatory networks in *P. patens* will greatly increase our understanding of plant tolerance to biotic and abiotic stresses.

## Figures and Tables

**Table 1:** *P. patens* and *A. thaliana* homologues of proteins involved in RNAi pathways (Updated after [81]).

Protein family	*P. patens* homologues	NCBI/Gene model number	*E*-value, % Identity	Molecular function	References
AtDCL1	PpDCL1a	EF670436	0.0, 68%	miRNA biogenesis Indispensable for target cleavage	[5,29,82,83]
	PpDCL1b	DQ675601	0.0, 65%		

AtDCL2	n.i 1	-	-	Generates endogenous siRNAs from a convergently transcribed and overlapping gene pairs Transitive silencing of transgenes Produces viral siRNAs	[74,84,85]

AtDCL3	PpDCL3	EF670437	1e^−116^, 32%	Generates siRNAs that guide chromatin modification in *P. patens* and *A. thaliana*	[21,69]

AtDCL4	PpDCL4	EF670438	1e^−124^, 33%	Generates trans-acting siRNAs (ta-siRNAs)	[54,55,57,60,86,87]

AtAGO1	PpAGO1a 2	Phypa_205541	0.0, 78%	Associates with the majority of miRNAs to guide the cleavage of their targets	[2,39,40,88,89]
	PpAGO1b 2	Phypa_158832	0.0, 77%		
	PpAGO1c 2	Phypa_141045	0.0, 75%		

AtAGO2	n.i	-	--	Known to be function in antiviral defense and ta-siRNAs biogenesis	[90,91]

AtAGO3	n.i	-	-	Not analyzed	

AtAGO4	PpAGO4^2^	Phypa_200513	1e^−164^, 38%	Involved in 24nt siRNA mediated gene silencing	[92–97]

AtAGO5	n.i	-	-	Not analyzed	

AtAGO6	PpAGO6^2^	Phypa_117253	1e^−152^, 39%	Involved in 24nt siRNA mediated DNA methylation	[93,98]

AtAGO7	n.i	-	-	Associates specifically with miR390 and directs cleavage of the *AtTAS3* precursor	[13–16,56,58]

AtAGO8	n.i	-	-	Not analyzed	

AtAGO9	PpAGO9^2^	Phypa_134255	6.1e^−160^, 40%	Preferentially interacts with 24nt siRNAs derived from transposable elements (TEs), required to silence TEs in female gametes and their accessory cells. Cell fate determination in the ovule.	[93,99]

AtAGO10	n.i	-	-	Implicated in miRNA-directed translational inhibition and repression of miR165/166 levels	[42,100,101]

AtRDR1	PpRDR1^2^	Phypa_219654	1.3e^−204^, 48%	Synthesis of long dsRNA from transgenes that can initiate different RNAi pathways Biogenesis of secondary siRNAs from RNA viruses	[102–104]

AtRDR2	n.i	-	-	Biogenesis of 24nt siRNAs from repeat loci involved in DNA methylation	[105,106]

AtRDR3a	n.i.	-	-	Not analyzed	[107,108]
AtRDR3b	PpRDR3b 2	Phypa_169723	1.8e^−96^, 33%		
AtRDR3c	PpRDR3c 2	Phypa_172848	6.1e^−89^, 31%		

AtRDR6	PpRDR6	Phypa_379	2.8e^−226^, 42%	Initiation and maintenance of dsRNA-induced RNAi in *A. thaliana* Conversion of *TAS* precursors into dsRNA in *P. patens* and *A. thaliana*	[12,14,109,110]

AtHEN1	PpHEN1^2^	Phypa_148777	3e^−56^, 33%	Methylates miRNA and siRNA duplexes at the 3′ end	[29–31,111]

AtHYL	PpHYL1^2^	Phypa_34761	7e^−31^, 50%	Interacts with AtDCL1 and confers stability to miRNA precursors	[69,112–114]

AtHASTY	PpHASTY1^2^	Phypa_137344	1.3e^−228^, 40%	Exports miRNA-miRNA * duplex to the cytoplasm	[14,48]
	PpHASTY2^2^	Phypa_151199	2.1e^−173^, 41%		

AtSE	PpSE1^2^	Phypa_133793	1.7e^−92^, 41%	Interacts with AtDCL1 and confers stability to miRNA precursors	[69,82,112,113]
	PpSE2^2^	Phypa_124567	1.8e^−70^, 41%		
	PpSE3^2^	Phypa_99415	3.3e^−53^, 35%		

AtCPL1	PpCPL1^2^	Phypa_432395	1e^−126^, 49%	Required for HYL1 dephosphorylation, which in turn is essential for accurate miRNA processing and strand selection.	[115]
	PpCPL2^2^	Phypa_429817	1e^−126^, 51%		

AtCBP20	PpCBP20a^2^	Phypa_442048	5e^−42^, 53%	Involved in pre-miRNA splicing and miRNA processing	[116,117]
	PpCBP20b^2^	Phypa_442049	7e^−71^, 58%		
	PpCBP20c^2^	Phypa_442050	7e^−69^,76%		

AtCBP80	PpCBP80.1^2^	Phypa_425787	0.0, 47%	Involved in pre-miRNA splicing and miRNA processing	[116,117]
	PpCBP80.2^2^	Phypa_432264	0.0, 47%		

AtSQN/CYP40	PpSQNa^2^	Phypa_433182	1e^−136^, 66%	Required for miRNA activity by promoting the activity of AGO1. Plays a unique and important role in plant RISC assembly	[118–120]
	PpSQNb^2^	Phypa_433181	1e^−136^, 66%		

AtHSP90	PpHsp90.1^2^	Phypa_456075	0.0, 80%	Plays a unique and important role in plant RISC assembly	[119,120]
	PpHsp90.2^2^	Phypa_454408	0.0, 80%		
	PpHsp90.3^2^	Phypa_452062	0.0, 79%		
	PpHsp90.4^2^	Phypa_452093	0.0, 80%		

AtSGS3	PpSGS3^2^	Phypa_448213	3.0e^−71^, 37%	Involved in the production of ta-siRNAs, through direct or indirect stabilisation of *TAS* cleavage products	[14,110]

AtPol IV	PpPol IV^2^	Phypa_132119	1.3e^−72^, 49%	Required for the biogenesis of 24nt siRNAs (with RDR2 and DCL3) that associate with AGO4 and direct DNA and histone modifications	[94–96]

AtPol V	PpPol V^2^	Phypa_129844	1e^−132^, 70%	Generates transcripts from heterochromatic regions (with DRD1) that are discussed to bind siRNA-AGO4 complexes directing DNA and histone modifications	[94–96]

AtDRM1	PpDRM1^2^	Phypa_148057	5e^−92^, 51%	Involved in the siRNA-directed *de novo* DNA methylation and maintenance of DNA methylation at CHH sites	[105,121,122]
AtDRM2	PpDRM2^2^	Phypa_133529	6.3e^−87^, 47%		

AtDRD1	PpDRD1^2^	Phypa_113504	1e^−109^, 35%	Cooperates with Pol V	[94,96,122–127]

AtSNF2	PpSNF2^2^	Phypa_211797	1.3e^−187^, 46%	Involved in the spreading of transgene silencing (with AtRDR2 and AtPol IV) and in the production of endogenous 24 nt siRNAs	[128–131]

AtRDM12	PpRDM12^2^	Phypa_98999	1e^−46^, 26%	Involved in the *de novo* DNA methylation and siRNA-mediated maintenance of DNA methylation	[132,133]

Protein sequences from *A. thaliana* (TAIR; available online: http://www.arabidopsis.org (accessed on 10 December 2012)) were used for reciprocal BLASTP searches against the *P. patens* V1.6_proteins database (available online http://www.cosmoss.org (accessed on 10 December 2012)).

1 Not identified.

2 Unknown function in *P. patens*.

DCL, Dicer Like; AGO, ARGONAUTE; RDR, RNA-Dependent RNA Polymerase; HEN1, Hua Enhancer 1, HYL1, Hyponastic Leaves 1; SE, Serrate; CPL1, *C*-Terminal Domain Phosphatase-like 1; CBP20, Cap-binding Protein 20; CBP80, Cap-binding Protein 80; SQN/CYP40, Squint/Cyclophilin 40; HSP90, Heat Shock Protein 90; SGS3, Suppressor of Gene Silencing 3; DRM, Domains Rearranged Methylase; DRD1, Defective in RNA-directed DNA Methylation 1; RDM12, RNA- directed DNA Methylation 12.
